# Expression deregulation of matrix metalloproteinases and vasoconstriction related genes in Pakistani females with abnormal uterine bleeding

**DOI:** 10.1186/s12905-022-02132-y

**Published:** 2022-12-23

**Authors:** Zertashia Akram, Ishrat Mahjabeen, Faiza Irshad, Malik Waqar Ahmed, Sadaf Rehman, Muhammad Rizwan, Amna Shafqat, Aniqa Kiran, Asma Saeed

**Affiliations:** Cancer Genetics and Epigenetics Lab, Department of Biosciences, COMSATS University Islamabad, Islamabad, Pakistan

**Keywords:** Abnormal uterine bleeding, Extracellular matrix, Menstruation, Miscarriages

## Abstract

**Background:**

Abnormal uterine bleeding (AUB) is irregular menstrual bleeding which has great impact on female health and life style. Various genetic factors are involved in etiology and pathology of AUB. Present study was designed to explore the association of PTGFR, MMP9, MMP2, TGFB3 and VEGFB with AUB.

**Methods:**

Blood samples of 212 females with AUB were collected along with age-matched healthy control. Expression variation of targeted genes was evaluated using qPCR. Present study cohort was divided into different groups based on demographic parameters and all targeted genes were correlated with study demographics.

**Results:**

Expression of targeted genes was significantly (*P* < 0.001) downregulated in females with AUB compared to control. Reduced (*P* < 0.01) expression of targeted genes was observed in all age groups (21–30, 31–40, 41–50 year) of AUB patients compared to respective control. Expression of VEGFB increased (*P* < 0.05) in AUB females with > 9 days bleeding compared to AUB patient had < 9 days bleeding. AUB women with miscarriage history showed upregulation in MMP2, TGFB3 (*P* < 0.05), and downregulation in MMP9 and VEGFB (*P* < 0.05) expression compared to AUB group with no miscarriage history. Expression of MMP2 increased (*P* < 0.05) in AUB females with > 60 kg body weigh compared to AUB patient with < 60 kg weight.

**Conclusion:**

Present study open a new window for diagnosis of AUB at early stages and suggested a possible involvement of PTGFR, MMP9, MMP2, TGFB3 and VEGFB as candidate biomarkers in AUB.

## Introduction

Normal menstrual cycle defines the time between first bleeding day from one cycle to the initiation of next cycle [10]. Abnormal uterine bleeding (AUB) defines irregular menstrual bleeding in which frequency, duration, or amount of blood from uterine corpus is excessive [16] or would be more than 7 days of period [56]. During climacteric phase, deregulation was observed in ovarian activity and in maturation of follicles which ultimately lead to AUB [53]. AUB is the most common clinical entity [54, 70], with global prevalence between 10 and 30% [39], in developing countries 8–27% and in Pakistan it is 11% [5]. Almost 70% gynecological problems in peri and post menopausal women are because of AUB. Limited data and use of various confused terminologies for the description of AUB has caused hindrance in interpretation of basic clinical research [74]. International Federation of Gynaecology and Obstetrics (FIGO) define AUB using PALM-COEIN classification in which PALM (Polyp, Adenomyosis, Leiomyoma, Malignant lesion) describes structural causes and COEIN (Coagulopathy, Ovulatory dysfunction, Endometrial dysfunction, Iatrogenic, Not yet classified) describes non-structural causes [54]. In addition, endocrine disorders like hypothyroidism, hyperprolactinemia and polycystic ovary syndrome are also considered as possible causes of AUB [49, 57].

A broad range of genetic factors are identified in etiology of AUB which regulate the process of menstruation and maintain the levels of different reproductive hormones and DNA mismatch repair alterations either directly or indirectly to sustain the endometrial integrity [77]. Prostaglandin F2 alpha (PGF_2α_) is a bioactive form of prostaglandins [62, 67] involved in luteolysis and restrict the production of progesterone responsible for vasodilation and vasoconstriction of endometrium [27]. PGF_2α_ is an active vasoconstrictor acted on smooth muscles lining in uterus, induce contraction of smooth muscles, reduces blood vessel caliber, provoke inflammatory response and pain [8, 64]. It is involved in regulation of cyclic changes of menstrual cycle. Clinically PGF_2α_ is abortifacient to induce labor or to terminate pregnancy, including missed or partial abortion [73].

Matrix metalloproteinases (MMPs), a family of extracellular matrix, all together these enzymes can break down the machinery of extracellular matrix, implicated in tissue remodeling [48]. Ability of MMPs to decompose extracellular matrix at natural pH makes them essential for endometrial shedding and regeneration during normal menstrual cycle [26]. Among MMP enzymes, almost more than 20 proteolytic enzymes could govern extracellular matrix breakdown in endometrium [7] and play their role in endometrial cell implantation [46]. Expression of many MMPs have been associated with different pathological states [75], like in tumor invasion and endometriotic tissues in endometriosis [11]. Numerous MMPs are expressed in uterine tissues during regular cycle. MMP-2 is detectable during all phases of menstrual cycle, expressed in glandular epithelial cells of endometrium both in proliferative or secretory phase [30]. Various tissue inhibitors of metalloproteinases (TIMP) are important regulators of MMP activity [15], like the activity of MMP-2 and MMP-9 is regulated by TIMP-1 [29]. Therefore, altered equilibrium between MMPs and TIMPs expression would affect the normal conduct of cell differentiation, growth resulted into aberrant ECM degradation [45, 69]. Additionally, MMPs has associated with other cell functions like release of growth factors controlling tissue restoration processes, and angiogenesis [32].

Process of angiogenesis is quite intense and pivotal for regeneration of damaged tissues, cyclic changes in ovaries, sewage clots, endometrial proliferation, embryonic and postnatal tissue growth [24, 73]. Vascular endothelial growth factor (VEGF) is a cytokine which regulate angiogenesis, increases vascular permeability and trigger the growth of endothelial cells [60]. It also bring out changes in extracellular matrix after binding specifically with endothelial cells [6]. In endometrium VEGFB functions as growth factor and differentiation factor in angiogenic process, promote endothelial cell proliferation and lesions of endometrium [43]. During menstruation, higher levels of various VEGF isoforms were observed in peritoneal fluid of females diagnosed with endometriosis [21]. TGFB family comprises a number of structurally and functionally similar secreted cytokines. TGFB3 plays its role in angiogenesis, cellular survival, differentiation, apoptosis and embryonic development, and it regulates the factors necessary for cell adhesion, matrix formation and important to avoid endometrial breakdown [20, 76]. TGFB signaling is required to regulate many reproductive processes in females like follicular growth and development, oocyte competence, ovulation, implantation, decidualization, uterine and embryonic development and pregnancy. Involvement of TGFB family in cell growth [65], cell motality, apoptosis, immune response and differentiation has been beneficial for the coordination of endometrial healing during menstruation [72].

Pakistan is a developing country and females are not much aware about the fact that how reproductive problems greatly affect not only their health but also quality of life and may contribute to family life issues as well [37]. Limited data has been reported so far to describe the genetic association of different genes in AUB pathology. Main objective of present study was to investigate the expression variation of MMP9, MMP2, PTGFR, VEGFB and TGFB3 in females with AUB disorder and comparison with healthy control females. In addition, to find the association between expression of targeted genes and different demographic parameters like age, marital status, body weight, miscarriage history and frequency of bleeding. Early and timely diagnosis of AUB is important for the betterment of women’s reproductive life, which ultimately improve their body health and generate great impact on quality of life.

## Materials and methods

### Study design

Present study was designed to investigate the association of different genes including PTGFR, MMP9, MMP2, VEGFB, TGFB3 with progress of abnormal uterine bleeding. Expression variation of targeted genes was estimated in AUB females and comparison with healthy control subjects. In addition, mRNA expression was correlated with different demographic parameters of current study including age, marital status, miscarriage history, body weight. Samples were collected with prior permission to each participant. Family history, medical history, routine life history, bleeding pattern, bleeding days and body weight was taken from each participant by a uniform questionnaire filled by them. Two main group were designed AUB patient group and healthy control group.

### Inclusion/exclusion criteria

Inclusion criteria were as follows: Females were included in AUB group who had complaint of abnormal and irregular uterine bleeding. Females had regular and normal menstrual cycle with no complaints related to menstruation were included in healthy control group. All participating females were between 21 and 50 years of age. Females diagnosed with any specific uterine disorder were excluded from the study. Women with < 21 year and > 50 year of age were excluded from the study.

### Ethical approval

Ethical approval was granted by Ethical Review Committee of COMSATS University Islamabad. Current study was performed according to principles of the Declaration of Helsinki.

### Sample collection

Blood samples of 212 females with AUB complaints were collected from different hospitals along with age matched control females with normal and regular menstrual cycle per month. Sample size of study was estimated by Sample Size Calculator (calculator.net) with 95% confidence interval and 5% margin of error. AUB females were divided into different sub-groups based on various demographic parameters like age, marital status, miscarriage history, bleeding duration and body weight.

### Expression analysis

RNA was extracted from whole blood of AUB and control subjects using Trizol reagent method [3]. Extracted RNA was quantified on Nanodrop spectrophotometer (ND-100, USA). PCR product was specified on 2% agarose gel by gel electrophoresis. β-actin was used as internal control. Primers of β-actin, PTGFR, MMP9, MMP2, VEGFB, TGFB3 and ACTB (housekeeping gene) were designed through IDT (Integrated DNA Technology). Coding sequences of mentioned genes were obtained from ensemble genome browser. The primer sequence of targeted genes was given in Table 1. Based on annealing temperature the reaction conditions were optimized for amplification of targeted genes. We used Quantitative Real Time PCR System (Applied Biosystem) to perform qPCR reaction. The comparative mRNA expression of PTGFR, MMP9, MMP2, VEGFB, TGFB3 and β-actin was estimated using 2^−delta delta CT^ analysis method. First we normalize the CT value of AUB patients and control with CT value of housekeeping gene (beta-actin) and than calculated the relative expression of above mentioned genes using the 2^−delta delta CT^ method.

**Table 1:** Primer sequence of PTGFR, MMP9, MMP2, VEGFB, TGFB3 and ACTB gene

Gene	Primer	Sequence	Product size (bp)
PTGFR	Forward	5′GAGAGGCATGGAGAAGAAACTC3′	105
PTGFR	Reverse	5′AGGGTGACATCATGGCAATAC3′	105
MMP9	Forward	5′CTGGAGACCTGAGAACCAATC3′	102
MMP9	Reverse	5′ATTTCGACTCTCCACGCATC3′	102
MMP2	Forward	5′TGATGGTGTCTGCTGGAAAG3′	89
MMP2	Reverse	5′CTACAGGACAGAGGGACTAGAG3′	89
VEGFB	Forward	5′GTGCTGTGAAGCCAGACA3′	119
VEGFB	Reverse	5′TGGAGTGGGATGGGTGAT3′	119
TGFB3	Forward	5′CAATGTGTCCTCAGTGGAGAA3′	99
TGFB3	Reverse	5′CTCTGCTCATTCCGCTTAGAG3′	99
ACTB	Forward	5′TTCTCTGACCTGAGTCTCCTT3′	116
ACTB	Reverse	5′ACACCCACAACACTGTCTTAG3′	116

### Statistical analysis

Statistical analysis was performed between control and AUB patient group using One-Way ANOVA, multiple comparison Tukey’s test, student’s t-test, X^2^ test and Spearman correlation analysis. Furthermore, comparison was made between different subgroups based on age, marital status, bleeding frequency, miscarriage history and body weight using Tukey’s test, student t-test and X^2^ test. Correlation between the expression variation of targeted genes including PTGFR, MMP9, MMP2, VEGFB and TGFB3 were analyzed using Spearman correlation analysis. One sample t-test was used to calculate the *P*-value of demographic parameter between AUB and control group. Data of both the groups was analyzed by GraphPad Prism8.0 version.

## Results

In present study cohort, 212 females with abnormal uterine bleeding (AUB) along with age matched controls were collected. The demographic details of AUB and control group is given in Table 2.

**Table 2:** Demographic details of AUB patients and control group of present study cohort

Parameters	AUB group	Control group	*P*-Value
Sample size	212	212	
Married	145 (68%)	132 (62%)	0.0299
Unmarried	67 (33%)	80 (40%)	0.0562
21–30 years	68 (34%)	70 (33%)	0.0092
31–40 years	88 (44%)	90 (42%)	0.0072
41–50 years	56 (26%)	52 (24%)	0.0236
With Miscarriages	75	45	0.1560
Without Miscarriages	70	87	0.0687

Value of *P* < 0.05 taken as level of significant. One sample *t*-test was used to analyze the data

### Relative expression of targeted genes

**Fig. 1:**
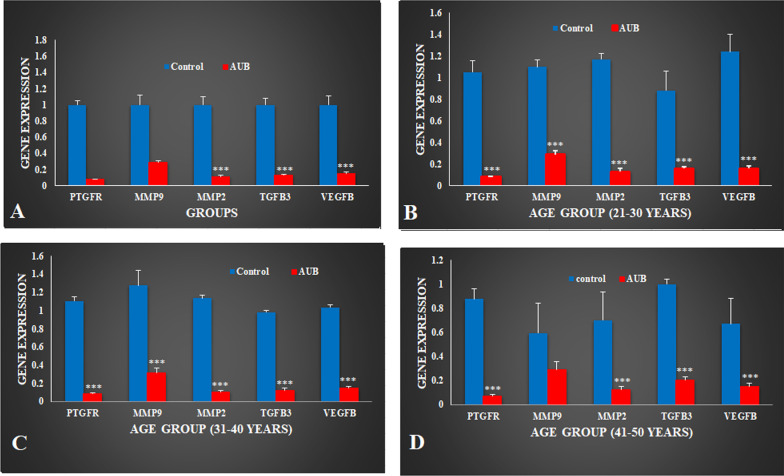
Relative expression of (n = 212/group) PTGFR, MMP9, MMP2, VEGFB and TGFB3 in **A** control and AUB females; **B** 21–30 year age group; **C** 31–40 year age group; **D** 41–50 year age group. Values are expressed as Mean ± SEM. Data was analyzed using one-way ANOVA and *P* value was calculated using student’s t-test. ***P < 0.001

Quantitative PCR was used to estimate the relative expression of our targeted genes in AUB females along with healthy control females. Relative expression of PTGFR (0.08 ± 0.005), MMP9 (0.28 ± 0.02), MMP2 (0.11 ± 0.01), VEGFB (0.15 ± 0.01) and TGFB3 (0.13 ± 0.08) (*P* < 0.001) reduced significantly in AUB females compared to healthy control females (1.00 ± 0.05, 1.00 ± 0.11, 1.00 ± 0.09, 1.00 ± 0.08, 1.00 ± 0.11) respectively (Fig. 1A).

### Age

Present study cohort of AUB and control females was divided into different groups based on age as 21–30 years, 31–40 years and 41–50 years. Expression variation was evaluated in targeted genes and comparison was made with respective controls.

Relative expression of PTGFR (0.07 ± 0.008), MMP9 (0.28 ± 0.03), MMP2 (0.12 ± 0.03), VEGFB (0.15 ± 0.02) and TGFB3 (0.15 ± 0.01) significantly (*P* < 0.001) downregulated in 21–30 years old AUB females compared to respective group (1.05 ± 0.11, 1.09 ± 0.06, 1.17 ± 0.05, 1.24 ± 0.16, 0.88 ± 0.18) of healthy control females (Fig. 1B). Likewise, expression of targeted genes (PTGFR 0.08 ± 0.008; MMP9 0.31 ± 0.05; MMP2 0.10 ± 0.01; TGFB3 0.12 ± 0.02; VEGFB 0.14 ± 0.01) reduced significantly (*P* < 0.001) in AUB females of 31–40 years age group compared to respective (1.10 ± 0.05, 1.28 ± 0.15, 1.13 ± 0.03, 0.98 ± 0.02, 1.03 ± 0.03) control group (Fig. 1C). Relative expression of PTGFR (0.07 ± 0.01), MMP2 (0.12 ± 0.02), VEGFB (0.15 ± 0.02) and TGFB3 (0.20 ± 0.02) reduced significantly (*P* < 0.001) in AUB females of 41–50 year age group compared to control females (0.87 ± 0.08, 0.70 ± 0.23, 0.67 ± 0.20, 1.00 ± 0.04) of same age respectively (Fig. 1D).

### Marital status

**Fig. 2:**
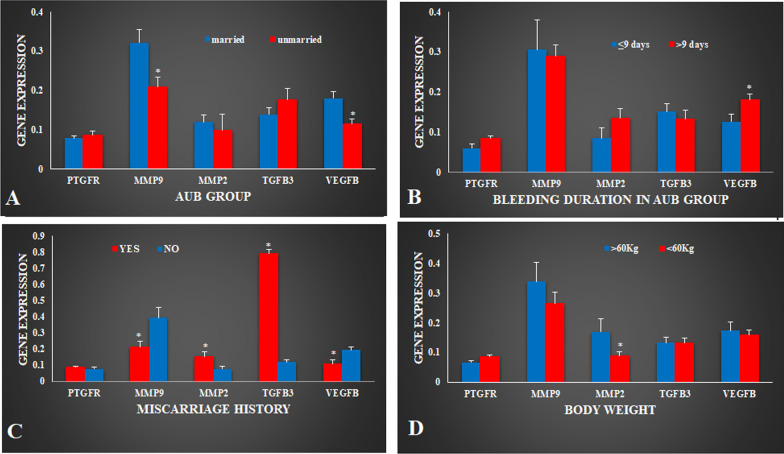
Relative expression of (n = 212/group) PTGFR, MMP9, MMP2, VEGFB and TGFB3 in AUB patients with **A** marital status; **B** bleeding duration; **C** miscarriage history; **D** body weight. Values are expressed as Mean ± SEM. Data was analyzed using one-way ANOVA and *P* value was calculated using student’s t-test.*P < 0.05

Study cohort of AUB patients was divided into two groups based on their marital status as married AUB and unmarried AUB female group. Relative expression of MMP9 (0.32 ± 0.03) and VEGFB (0.18 ± 0.01) was significantly (*P* < 0.05) upregulated in AUB married group compared to AUB unmarried (0.21 ± 0.02, 0.11 ± 0.01) females. While expression of PTGFR (0.07 ± 0.005), MMP2 (0.11 ± 0.01) and TGFB3 (0.13 ± 0.01) showed non-significant difference between married and unmarried (0.08 ± 0.01, 0.09 ± 0.04, 0.17 ± 0.03) AUB females respectively (Fig. 2A).

### Duration of bleeding

AUB group of study cohort was divided into two sub-groups based on duration of bleeding. One group comprised females bleed for < 9 days and the other group of females bleed for > 9 days. Relative expression of VEGFB (0.18 ± 0.01) gene significantly (*P* < 0.05) upregulated in AUB group with > 9 days of bleeding compared to AUB females with < 9 days (0.12 ± 0.01) of bleeding. Similar pattern was observed in expression of PTGFR (0.06 ± 0.01, 0.08 ± 0.006) and MMP2 (0.08 ± 0.02, 0.13 ± 0.02) gene in AUB group with > 9 days of bleeding although the difference was statistically non-significant (Fig. 2B).

### Miscarriage history

AUB females were divided into two groups based on their miscarriage history as females with history of miscarriage, and AUB females with history of no miscarriage. Relative expression of MMP2 (0.15 ± 0.01) and TGFB3 (0.79 ± 0.02) increased significantly (*P* < 0.01) in AUB females with miscarriage history compared to AUB females with no miscarriage (0.07 ± 0.01, 0.11 ± 0.01) history respectively. In contrast, expression of MMP9 (0.21 ± 0.03) and VEGF (0.10 ± 0.02) decreased significantly (*P* < 0.05) in AUB females with miscarriage compared to AUB females with no (0.39 ± 0.06, 0.19 ± 0.01) miscarriage history. No significant difference was observed in expression of PTGFR (0.08 ± 0.008, 0.07 ± 0.01) between two groups (Fig. 2C).

### Body weight

Present study cohort of AUB females were divided into two group based on their body weight as females with ≤ 60 kg body weight and > 60 kg body weight. Relative expression of MMP2 (0.16 ± 0.04) gene was significantly (*P* < 0.05) upregulated in AUB females with > 60 kg body weight compared to females with < 60 kg (0.08 ± 0.01) body weight. However other targeted genes (PTGFR 0.06 ± 0.006, 0.08 ± 0.007; MMP9 0.33 ± 0.06, 0.26 ± 0.03; TGFB3 0.13 ± 0.02, 0.13 ± 0.01; VEGFB 0.17 ± 0.02, 0.16 ± 0.01) showed non-significant expression variation between two groups of AUB females (Fig. 2D).

### Correlation between targeted genes

**Fig. 3:**
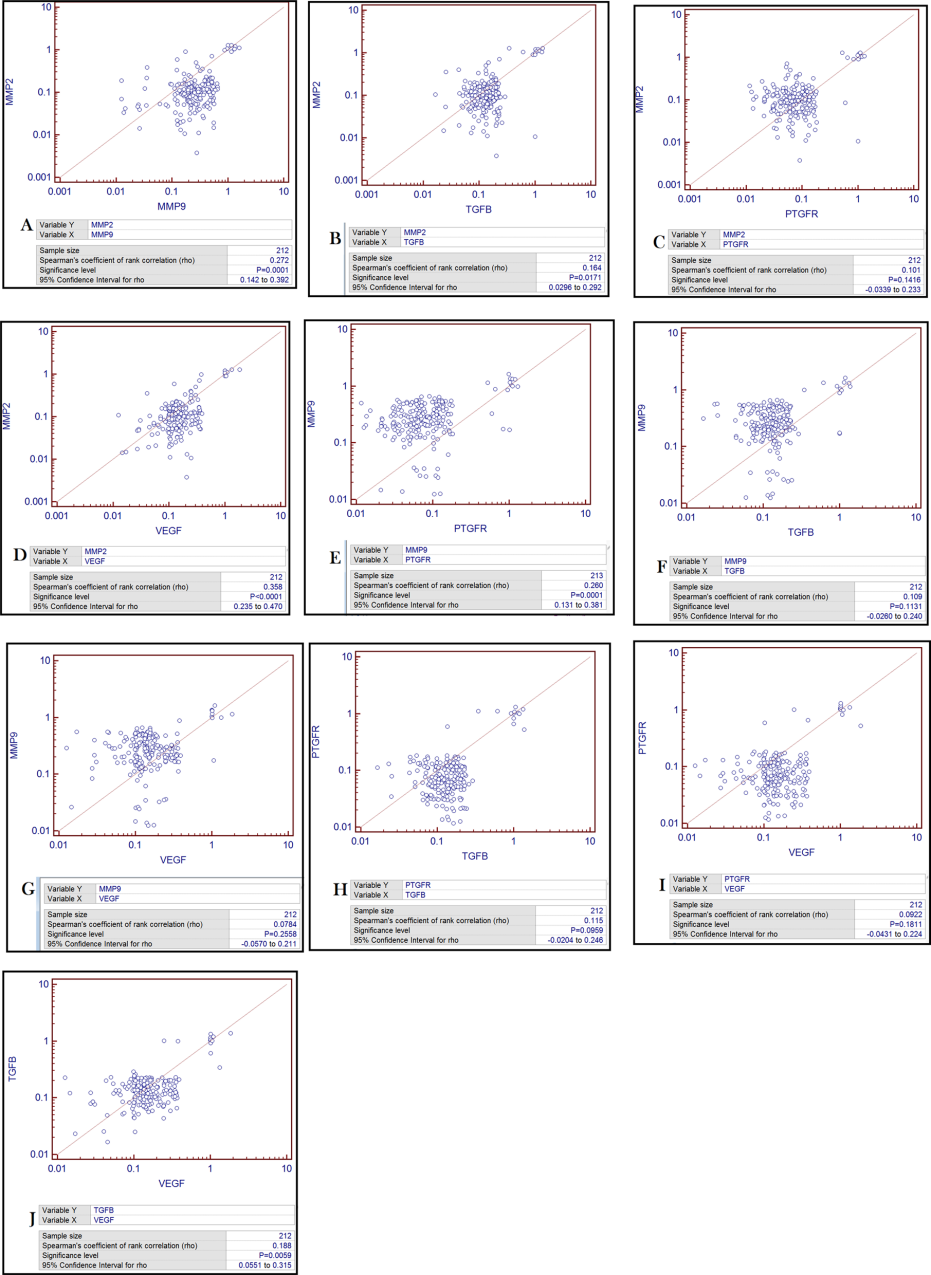
Spearman’s correlation of mRNA expression of targeted genes PTGFR, MMP9, MMP2, VEGFB and TGFB (n = 212/group) with one another. Rho = Spearman's coefficient; level of significance = *P* < 0.05; **A** = MMP2 versus MMP9; **B** = MMP2 versus TGFB; **C** = MMP2 versus PTGFR; **D** = MMP2 versus VEGF; **E** = MMP9 versus PTGFR; **F** = MMP9 versus TGFB; **G** = MMP9 versus VEGF; **H** = PTGFR versus TGFB; **I** = PTGFR versus VEGF; **J** = TGFB versus VEGF

Spearman’s correlation analysis was done to find the association between five focused genes of present study. Expression variation of five genes including PTGFR, MMP9, MMP2, VEGFB and TGFB3 were compared to one another and we observed a positive correlation between all genes (Fig. 3). Significant positive correlation was found between MMP2 vs MMP9 (r = 0.272; *P* = 0.0001; (Fig. 3A), MMP2 vs TGFB3 (r = 0.164; *P* = 0.0171; (Fig. 3B), MMP2 vs VEGFB (r = 0.358, *P* = 0.0001; (Fig. 3D), MMP9 vs PTGFR (r = 0.260, *P* = 0.0001; (Fig. 3E), TGFB3 vs VEGF (r = 0.188; *P* = 0.0059; (Fig. 3J). Whereas, positive but statistically non-significant correlation was observed between MMP2 vs PTGFR (r = 0.101; *P* = 0.1416; (Fig. 3C), MMP9 vs TGFB3 (r = 0.109; *P* = 0.1131; (Fig. 3F), MMP9 vs VEGFB (r = 0.0784; *P* = 0.2558; (Fig. 3G), PTGFR vs TGFB3 (r = 0.115; *P* = 0.0959; (Fig. 3H), PTGFR vs VEGFB (r = 0.0922; *P* = 0.1811; (Fig. 3I).

## Discussion

Women health issues are being ignored in our society especially in developing countries. Reproductive health in particular has not been addressed properly. Generally, females of developing countries do not understand the negative impact of poor reproductive/menstrual health on all aspects of their life including life quality, emotions, mental and body health and even their relationships (Varsha et al. 2022). Menstrual health has been hindered due to lack of understanding about the underlying mechanism of uterine physiology and menstrual cycle [18, 19], although one in four women is suffering from heavy uterine bleeding (Hilary et al. 2020). Not all women menstruate in normal/regular fashion and the prevalence of menstrual disorder become increase in latter part of twentieth century [54] Therefore, abnormal uterine bleeding (AUB) is one of the common female gynecological disorder, unfortunately taken as normal routine life change in women’s reproductive health. Present study was designed to evaluate the possible involvement of five different genes (PTGFR, MMP9, MMP2, VEGFB, TGFB3) in prognosis of abnormal uterine bleeding and their association with different demographic parameters.

Matrix metalloproteinases (MMPs) are proteolytic enzymes excreted by different pro-inflammatory cells, involved in degradation of extracelluar matrix (ECM) and play an important role in menstruation [11]. MMPs balance plays a critical role in physiological process and pathological conditions such as tissue repair, angiogenesis, trophoblast invasion, wound healing, tumor growth and menstruation [33, 34, 46]. In present study we observed significant downregulation in MMP9 and MMP2 mRNA expression in blood samples of females suffering with AUB compared to women with normal menstrual cycle and had a regular bleeding pattern. Expression of MMP9 was lower in thin endometrium with < 7 mm thickness compared to endomterium with > 7 mm thickness (Li et al. 2022). MMP9 and MMP2 have been localized in endometrial tissue and their high expression was detected during menstrual phase compared to other phases of cycle [46]. There is an association between low plasma levels of MMP9 and decreased levels of endothelial inhibitor angiostatin in terms of increased tumor growth and vascularization [58]. Different researchers observed the association of MMP9 secretion with menstruation, endometrium remodeling and ovulation [14, 22, 35]. In present study expression of MMP9 and MMP2 remained significantly low in AUB females of different age groups compared to respective control females. Whereas, increase expression of MMP9 and MMP2 gene was observed in AUB females with > 60 kg body weight compared to AUB females with < 60 kg body weight. Menstrual disorder are more common in overweight women compared to females with normal body mass index (BMI) [61]. Significant increase in serum levels of MMP9 was reported in obese females compared to women of normal BMI [28]. Obese female have high levels of free testosterone which is not converted to estradiol through aromatase enzymes [61], may act as one possible factor to deregulate MMPs expression. In contrast, females with low BMI also complaint menstrual disturbance might be due to altered leptin signaling [71]. Altogether, these results would suggest the possible influence of body mass on expression of MMPs in women with abnormal uterine bleeding and open a new window in context with association of BMI with abnormal uterine bleeding. Present study showed upregulation of MMP9 in married AUB females compared to unmarried AUB females. In late secretory phase there is withdrawal in progesterone levels which is an anti-inflammatory hormone, therefore the increase in production of chemokines, cytokines and MMPs is evident [25]. Inhibited MMPs activity in response to increased levels of progesteron would maintain secretory endometrium. The case is revert after the withdrawal of progesterone levels and afterward a threshold will come when endometrium is no more receptive to anti-inflammatory activities of progesterone [9, 47]. Any deregulation/disruption in this pathway would lead to AUB [54]. Possibly, in married AUB females the progesterone imbalance may activate the abnormal behavior of MMPs resulted in heavy uterine bleeding. Married womens have high levels of steroid hormone compared to unmarried females [12]. Unfortunately, in present study we have not estimated the levels of hormones. For future research, it would be suggested to up look the serum levels of reproductive hormones and their stimulators as well.

Reproductive organs are unique in nature particularly human endometrium which gone through number of repetitive cyclic changes of cell proliferation, differentiation, remodeling and repair after every 28 days. These changes have been regulated by interaction of different hormones, cytokines and angiogenic growth factors [59]. Endometrium as enriched source of angiogenic factors, showing expression variations throughout the menstrual cycle [41]. Transforming growth factor (TGFB) family strongly influence the fertility and reproductive functions of different organisms [52]. In present study, expression of TGFB3 was decreasedsignificantly in AUB patients compared to respective control females. Moreover, females of different age groups had shown reduced levels of TGFB3 compared to healthy female group. Reported data has suggested the possible association of aberrant vascular maturity to heavy menstrual bleeding (HMB) and recurrent pregnancy loss [44]. Reduced expression of TGFβ1 was observed in women with heavy menstrual bleeding [50]. TGFB playing its role in tumor progression by stimulating tumor growth and metabolism and is one of the key element involved in pathology of uterine fibroid [17]. Low expression and down signaling of TGFB1 was observed in endometrium of female diagnosed with heavy menstrual bleeding [44]. TGFB involved in vascular growth directly by interacting with extra cellular matrix (ECM). Dysregulation of any factor in ECM may alter the expression of TGFB, probably leading to failure in mature angiogenesis hence inducing extensive bleeding and menstrual shedding. This would support the results of current study where we observed decreased expression of MMP9, MMP2 along with downregulation of TGFB.

Expression variation of VEGF and reproductive anomalies like recurrent miscarriage and implantation failure are correlated with each other [31, 38]. Moreover, VEGF is involved in tumor growth and metastasis [6]. We observed reduced expression of VEGFB in AUB females compared to healthy females. Similar pattern of downregulation was noticed in different age groups of AUB females compared to control females of respective age. Risk of AUB increases with age [1]. Interestingly, VEGFB levels were higher in married AUB females compared to unmarried AUB females. VEGF expressed in theca cells and granulosa cells of follicles in ovarian tissue [2]. These cells release reproductive hormone (estrogen, androgen, progesterone) under the stimulation of FSH and LH from pituitary gland. Possibly altered levels of any one of these hormones may variate the VEGF expression in married females compared to unmarried females. As levels of estradiol and progesterone are higher in married females compared to unmarried women [12]. Likewise, same increase was observed in AUB females experienced > 9 days of bleeding compared to AUB females with < 9 days of bleeding. Angiogenic factor like VEGF play their role for normal function of female reproductive system [63]. Sufficient levels of VEGF are required to accomplish successful implantation/pregnancy [41]. Expression variation in VEGF and reproductive failure like recurrent miscarriages are correlated to one another [31]. Likewise, in present study VEGFB expression was lower in AUB females with previous history of miscarriages compared to AUB females with no history of miscarriages. VEGF expression variation was observed in females with recurrent miscarriages [4]. (To date efforts have made to clarify the exact role of VEGF in reproduction and implantation but unfortunately, limited data availability restrict the researchers to interpret the exact role of VEGF in AUB disorder although, VEGF is documented as angiogenic factor playing its role in different pathological conditions [31]. During menstruation, endometrium is like wounded mucosa need rapid repair onmonthly basis (Hilary et al. 2020). Any small increase in vessel diameter would greatly reduce the resistance to blood flow [18, 19] and increase the blood flow in vessels. Possibly altered levels of VEGFB may restrict angiogenesis, enhance uterine bleeding in AUB women, and suppress immune response of body, which facilitate the inflammation in AUB females.

Prostaglandins are well known for their importance in female reproduction [68] including implantation, ovulation and uterine maintenance [36, 40]. In uterine tissue prostaglandins work through different receptors and its function depends on expression of receptors [13]. PGF_2α_, is regulated by PTGFR receptor and this binding regulate the uterine contraction, promote luteolysis and help in parturition [23]. In our study PTGFR expression was downregulated in AUB females compared to healthy females with normal menstrual cycle. The decreased pattern was continue in females of all age groups. During menstruation, PGF_2α_ acted as local vasoconstrictor in endometrium of uterus. Withdrawal of progesterone may decrease the expression of vasoconstriction factor like PGF_2α_ and PTGFR along with decreased levels of endothelin 1 (potent vasoconstrictor) play their role in AUB disorder [51]. Knock out of FP receptors in females mice results in loss of parturition [55]. Similarly, FP receptor gene was downregulated in myometrium and endometrium of bitches affected with pyometra [66]. Downregulation of PTGFR may reduce the vasoconstriction ability of arteriole in endometrium, lead to increase blood loss resulted into abnormal uterine bleeding or it may increase the bleeding duration as well. Reduced muscle cell proliferative activity reported in spiral arteriole of women with heavy bleeding compared to females with normal bleeding [51].

Present study provide a baseline mechanism for upcoming research to explore the association of MMP2, MMP9, VEGFB, PTGFR and TGFB3 in pathogenesis of abnormal uterine bleeding. Matrix metalloproteinases may initiate damage to myometrium. VEGF involved in menstruation and endometrial repair, and angiogenesis is integral part to repair process during menstruation. VEGF would promote increase bleeding in AUB females. TGF monitor formation of extracellular matrix (ECM), vascular growth and cell adhesion. MMPs deregulation alter the expression of TGFB that would affect cellular adhesion and may degrade cellular matrix, which ultimately initiate abnormal bleeding from uterine corpus. In addition to genetic factors, age, marital status, miscarriages and body weight have all play their role in AUB progression. Age, reduces the contractile ability of smooth muscle cells in uterine lining, which reduce the resistance against blood loss. Recurrent miscarriages and marital status may alter levels of reproductive hormone, which deregulate the expression of targeted genes. The targeted genes acted as a marker of AUB therefore, present study will be helpful in diagnosis of abnormal uterine bleeding disorder at early stage and benefit not only the female reproductive and body health but their mental health as well.

### Study limitation and strength

PTGFR, MMPs, VEGF and TGFB genes are important contributor in process of menstruation, playing their role in ECM remodeling, angiogenesis and vasoconstriction of uterine tissue. It is worth important to evaluate the expression deregulation of these genes. So far, limited data reported the consequences of AUB in association with targeted genes. Present study address the molecular cause of AUB pathogenesis and will open a new baseline window for targeted genes to act as biomarkers for the early diagnosis of AUB. This would improve the women reproductive health, body health and mental health as well.

There were various limitation in current study. First limitation is small study cohort and to obtain detailed and clear picture of underlying mechanism of PTGFR, MMP9, MMP2, VEGFB, TGFB genes large study cohort would be suggested. Secondly, our major target was to evaluate the mRNA expression variation in targeted genes of AUB patients and we do not focused on the levels of steroid and pituitary hormones that regulate the process of uterine bleeding. Correlation between hormone levels and these genes would be good future finding. Thirdly, future studies should consider environmental factors, parity, diet, caesarean section, exercise and drug usage for complete consequences of AUB underlying mechanism. In addition, we targeted at mRNA level of genes but in future western blot assay and ELISA would be suggested to take the translational level of targeted genes. Due to unavailability of tissue biopsies we choose the blood samples.

## Data Availability

All data generated and/or analyzed during current study is included in this article.

## References

[1] Abbott JA (2017). Adenomyosis and abnormal uterine bleeding (AUB-A)–pathogenesis, diagnosis, and management. Best Pract Res Clin Obstet Gynaecol.

[2] Abdel-Ghani MA, Shimizu T, Suzuki H (2014). Expression pattern of vascular endothelial growth factor in canine folliculogenesis and its effect on the growth and development of follicles after ovarian organ culture. Reprod Domest Anim.

[3] Ahmad S, Arif B, Akram Z, Ahmed MW (2020). Association of intronic polymorphisms (rs1549339, rs13402242) and mRNA expression variations in PSMD1 gene in arsenic-exposed workers. Environ Sci Pollut Res.

[4] Amirchaghmaghi E, Rezaei A, Moini A, Roghaei MA, Hafezi M, Aflatoonian R (2015). Gene expression analysis of VEGF and its receptors and assessment of its serum level in unexplained recurrent spontaneous abortion. Cell J.

[5] Ansari A, Urooj U (2020). Study of causes behind abnormal uterine bleeding according to PALM-COEIN classification at a tertiary care hospital. JPMA.

[6] Apte RS, Chen DS, Ferrara N (2019). VEGF in signaling and disease: beyond discovery and development. Cell.

[7] Apte SS, Parks WC (2015). Metalloproteinases: a parade of functions in matrix biology and an outlook for the future. Matrix Biol.

[8] Arif R, Mazhar T, Jamil M (2019). Induction of labor in primigravid term pregnancy with misoprostol or dinoprostone: a comparative study. Cureus.

[9] Armstrong GM, Maybin JA, Murray AA, Nicol M, Walker C, Saunders PTK, Rossi AG, Critchley HOD (2017). Endometrial apoptosis and neutrophil infiltration during menstruation exhibits spatial and temporal dynamics that are recapitulated in a mouse model. Sci Rep.

[10] Baker FC, Lee KA (2018). Menstrual cycle effects on sleep. Sleep Med Clin.

[11] Balkowiec M, Maksym RB, Wlodarski PK (2018). The bimodal role of matrix metalloproteinases and their inhibitors in etiology and pathogenesis of endometriosis (Review). Mol Med Rep.

[12] Barrett ES, Tran V, Thurston AW, Frydenberg H, Lipson SF, Thune I, Ellison PT (2015). Women who are married or living as married have higher salivary estradiol and progesterone than unmarried women. Am J Hum Biol.

[13] Blesson C, Buttner E, Masironi B, Sahlin L (2012). Prostaglandin receptors EP and FP are regulated by estradiol and progesterone in the uterus of ovariectomized rats. Reprod Biol Endocrinol.

[14] Bourdon G, Cadoret V, Charpigny G (2021). Progress and challenges in developing organoids in farm animal species for the study of reproduction and their applications to reproductive biotechnologies. Vet Res.

[15] Cabral-Pacheco GA, Garza-Veloz I, la Rosa CC, Ramirez-Acuña JM, Perez-Romero BA, Guerrero-Rodriguez JF, Martinez-Avila N, Martinez-Fierro ML (2020). The roles of matrix metalloproteinases and their inhibitors in human diseases. Int J Mol Sci.

[16] Chodankar R, Critchley HO (2019). Biomarkers in abnormal uterine bleeding. Biol Reprod.

[17] Ciebiera M, Włodarczyk M, Wrzosek M, Eczekalski BM, Nowicka G, Łukaszuk KM (2017). Role of transforming growth factor β in uterine fibroid biology. Int J Mol Sci.

[18] Critchley HOD, Babayev E, Bulun SF, Clark S, Garcia-Grau I, Gregersen PK, Kilcoyne A, Kim JYJ, Lavender M, Marsh EE, Matteson KA (2020). Menstruation: science and society. Am J Obstet Gynecol.

[19] Critchley HOD, Maybin JA, Armstrong GM, Williams ARW (2020). Physiology of the endometrium and regulation of menstruation. Physiol Rev.

[20] Cruz CD, Reis FM (2015). The role of TGFβ superfamily members in the pathophysiology of endometriosis. Gynecol Endocrinol.

[21] Danastas K, Miller EJ, Hey-Cunningham AJ, Murphy CR, Lindsay LA (2018). Expression of vascular endothelial growth factor A isoforms is dysregulated in women with endometriosis. Reprod Fertil Dev.

[22] De Bem THC, Tinning H, Vasconcelos EJR (2021). Endometrium on-a-chip reveals insulin- and glucose-induced alterations in the transcriptome and proteomic secretome. Endocrinology.

[23] Durn JH, Marshall KM, Farrar D (2010). Lipidomic analysis reveals prostanoid profiles in human term pregnant myometrium. Prostaglandins Leukotrienes Essential Fatty Acids.

[24] Elfayomy AK, Almasry SM, Attia GM, Habib FA (2015). Enhanced expression of vascular endothelial growth factor and increased microvascular density in women with endometrial hyperplasia: a possible relationship with uterine natural killer cells. Rom J Morphol Embryol.

[25] Evans J, Salamonsen LA (2014). Decidualized human endometrial stromal cells are sensors of hormone withdrawal in the menstrual inflammatory cascade. Biol Reprod.

[26] Goffin F, Munaut C, Frankenne F, d’Hauterive PS, Beliard A, Fridman V, Nervo P, Colige A, Foidart JM (2003). Expression pattern of metalloproteinases and tissue inhibitors of matrix-metalloproteinases in cycling human endometrium. Biol Reprod.

[27] Goravanahally MP, Sen A, Inskeep EK, Flores JA (2007). PKCepsilon and an increase in intracellular calcium concentration are necessary for PGF2alpha to inhibit LH-stimulated progesterone secretion in cultured bovine steroidogenic luteal cells. Reprod Biol Endocrinol.

[28] Grzechocinska B, Dąbrowski F, Sierdzinski J, Cyganek A, Wielgos M (2019). The association between serum metalloproteinase concentration, obesity, and hormone levels in reproductive-aged women. Endokrynol Pol.

[29] Grzechocinska B, Dabrowski FA, Chlebus M, Gondek A, Czarzasta K, Michalowski L, Cudnoch-Jedrzejewska A, Wielgos M (2018). Expression of matrix metalloproteinase enzymes in endometrium of women with abnormal uterine bleeding. Neuro Endocrinol Lett.

[30] Grzechocinska F, Dąbrowski A, Cyganek GP, Miroslaw W (2017). The role of metalloproteinases in endometrial remodelling during menstrual cycle. Polish Gynecolo.

[31] Guo X, Yi H, Li TC, Wang Y, Wang H, Chen X (2021). Role of vascular endothelial growth factor (VEGF) in human embryo implantation: clinical implications. Biomolecules.

[32] Haibe Y, Kreidieh M, ElHajj H, Khalifeh I, Mukherji D, Temraz S, Shamseddine A (2020). Resistance mechanisms to anti-angiogenic therapies in cancer. Front Oncol.

[33] Hamutoglu R, Bulut HE, Kaloglu C, Onder O, Dagdeviren T, Aydemir MN, Korkmaz EM (2020). The regulation of trophoblast invasion and decidual reaction by matrix metalloproteinase-2, metalloproteinase-7, and metalloproteinase-9 expressions in the rat endometrium. Reprod Med Biol.

[34] Hsu HH, Hu WS, Lin YM, Kuo WW, Chen LM, Hwang JM, Tsai FJ, Liu CJ, Huang CY (2011). JNK suppression is essential for 17β-Estradiol inhibits prostaglandin E2-Induced uPA and MMP-9 expressions and cell migration in human LoVo colon cancer cells. J Biomed Sci.

[35] Hu S, Wang Y, Ke D (2018). Antifertility effectiveness of a novel copper-containing intrauterine device material and its influence on the endometrial environment in rats. Mater Sci Eng C Mater Biol Appl.

[36] Jabbour HN, Sales KJ (2004). Prostaglandin receptor signalling and function in human endometrial pathology. Trends Endocrinol Metabol.

[37] Jain V, Chodankar RR, Maybin JA, Critchley HOD (2022). Uterine bleeding: how understanding endometrial physiology underpins menstrual health. Nat Rev Endocrinol.

[38] Jee BC, Suh CS, Kim KC, Lee WD, Kim H, Kim SH (2009). Expression of vascular endothelial growth factor-A and its receptor-1 in a luteal endometrium in patients with repeated in vitro fertilization failure. Fertil Steril.

[39] Kazemijaliseh H, Ramezani TF, Behboudi-Gandevani S, Hosseinpanah F, Azizi F (2017). A Population-based study of the prevalence of abnormal uterine bleeding and its related factors among iranian reproductive-age women: an updated data. Arch Iran Med.

[40] Kumar R, Ramteke PW, Nath A, Pramod RK, Singh SP, Sharma SK, Kumar S (2013). Role of candidate genes regulating uterine prostaglandins biosynthesis for maternal recognition of pregnancy in domestic animals. Inter Scholarly Res Not.

[41] Lash GE, Innes BA, Drury JA, Robson SC, Quenby S, Bulmer JN (2012). Localization of angiogenic growth factors and their receptors in the human endometrium throughout the menstrual cycle and in recurrent miscarriage. Hum Reprod.

[42] Lin-Hong L, Shi G, Jin-Bing P, Cai-Hong W, Zhao M, Xiu-Ping Z (2022). The expressions of matrix metalloproteinase-9, estrogen receptor, and progesterone receptor in thin endometrial tissue and their significance. Gynecol Endocrinol.

[43] Liu Q, Li Y, Zhao J, Sun DL, Duan YN, Wang N (2009). Association of polymorphisms -1154G/A and -2578C/A in the vascular endothelial growth factor gene with decreased risk of endometriosis in Chinese women. Hum Reprod.

[44] Lu Q, Sun D, Shivhare SB, Hou H, Bulmer JN, Innes BA, Hapangama DK, Lash GE (2021). Transforming growth factor (TGF) β and endometrial vascular maturation. Front Cell Dev Biol.

[45] Luddi A, Gori M, Marrocco C, Capaldo A, Pavone V, Bianchi L, Boschi L, Morgante G, Piomboni P, de Leo V (2018). Matrix metalloproteinases and their inhibitors in human cumulus and granulosa cells as biomarkers for oocyte quality estimation. Fertil Steril.

[46] Madjid TH, Ardiansyah DF, Permadi W, Hernowo B (2020). Expression of matrix metalloproteinase-9 and tissue inhibitor of metalloproteinase-1 in endometriosis menstrual blood. Diagnostics.

[47] Martínez-Aguilar R, Kershaw LE, Reavey JJ, Critchley HO, Maybin JA (2021). Hypoxia and reproductive health: the presence and role of hypoxia in the endometrium. Reproduction.

[48] Martins JMA, Rabelo-Santos SH, do Amaral, W.M.C., Zeferino, L.C.,  (2020). Tumoral and stromal expression of MMP-2, MMP-9, MMP-14, TIMP-1, TIMP-2, and VEGF-A in cervical cancer patient survival: a competing risk analysis. B.M.C. Cancer.

[49] Masylyanskaya S, Talib HJ, Northridge JL, Jacobs AM, Coble C, Coupey SM (2017). Polycystic ovary syndrome: an under-recognized cause of abnormal bleeding in adolescents admitted to children’s hospital. J Pediatr Adolesc Gynecol.

[50] Maybin JA, Boswell L, Young VJ, Duncan WC, Critchley HO (2017). Reduced transforming growth Factor-β activity in the endometrium of women with heavy menstrual bleeding. J Clin Endocrinol Metab.

[51] Mints M, Hultenby K, Zetterberg E, Blomgren B, Falconer C, Rogers R, Palmblad J (2007). Wall discontinuities and increased expression of vascular endothelial growth factor-A and vascular endothelial growth factor receptors 1 and 2 in endometrial blood vessels of women with menorrhagia. Fertil Steril.

[52] Monsivais D, Matzuk MM, Pangas SA (2017). The TGF-b family in the reproductive tract. Cold Spring Harb Perspect Biol.

[53] Munro GM, Critchley HO, Fraser IS (2012). The FIGO systems for nomenclature and classification of causes of abnormal uterine bleeding in the reproductive years: who needs them?. A.J.O.G..

[54] Munro MG, Critchley HOD, Fraser IS (2018). FIGO Menstrual Disorders Committee. The two FIGO systems for normal and abnormal uterine bleeding symptoms and classification of causes of abnormal uterine bleeding in the reproductive years. Int J Gynaecol Obstet.

[55] Narumiya S, FitzGerald GA (2001). Genetic and pharmacological analysis of prostanoid receptor function. J Clin Investig.

[56] Neelgund S, Hiremath PB (2016). Abnormal uterine bleeding in perimenopause. J Evol Med Dental Sci.

[57] Nur Azurah AG, Sanci L, Moore E, Groover S (2013). The quality of life of adolescents with menstrual problems. J Pediatr Adolesc Gynecol.

[58] Pozzi A, Wendy F, Humphrey AG (2002). Low plasma levels of matrix metalloproteinase 9 permit increased tumor angiogenesis. Oncogene.

[59] Qinsheng LU, Sun D, Shivhare SB, Hou H, Bulmer JN, Innes BA, Hapangama DK, Lash GE (2021). Transforming Growth Factor (TGF) β and endometrial vascular maturation. Front Cell Dev Biol.

[60] Rajuddin R, Oscar R, Dewi TP (2020). Serum vascular endothelial growth factor levels and uterine fibroid volume. Indones J Obstet Gynecol..

[61] Reavey, J.J., Duncan, W.C., Brito-Mutunayagam, S., Reynolds, R.M., Critchley, H.O.D., 2020. in Obesity and Gynecology 2nd edn Ch. 19 (eds Mahmoud, T., Arulkumaran, S. & Chervenak, F.) 171–177 (Elsevier, 2020).

[62] Ricciotti E, FitzGerald GA (2011). Prostaglandins and inflammation. A.T.V.B..

[63] Rizov M, Andreeva P, Dimova I (2017). Molecular regulation and role of angiogenesis in reproduction Taiwanese. J Obstet Gynecol.

[64] Robeldo T, Canzi EF, de Andrade PM, Santana JPP, Teixeira FR, Spagnol V, Lameiro BH, Maia NS (2020). Effect of tahiti lime (citrus latifolia) juice on the production of the PGF2α/PGE2 and pro-inflammatory cytokines involved in menstruation. Sci Rep.

[65] Schmierer B, Hill CS (2007). TGFβ–SMAD signal transduction: molecular specificity and functional flexibility. Nat Rev Mol Cell Biol.

[66] Shyamkumar TS, Kesavan M, Parida S, Patra MK, Panigrahi M, Mathesh K, Gokul C, Aneesha VA, Suhas KS, Kumar D, Telang AG (2022). Differential expression of prostaglandin receptors in canine uterus with pyometra. Top Companion Anim Med.

[67] Sriprasert I, Pakrashi T, Kimble T, Archer DF (2017). Heavy menstrual bleeding diagnosis and medical management. Contracep Reprod Med.

[68] Sugimoto Y, Inazumi T, Tsuchiya S (2015). Roles of prostaglandin receptors in female reproduction. J Biochem.

[69] Sundrani D, Narang A, Mehendale S, Joshi S, Chavan-Gautam P (2017). Investigating the expression of MMPs and TIMPs in preterm placenta and role of CpG methylation in regulating MMP-9 expression. IUBMB Life.

[70] Talukdar B, Mahela S (2016). Abnormal uterine bleeding in perimenopausal women: correlation with sonographic findings and histopathological examination of hysterectomy specimens. J Midlife Health.

[71] Thong EP, Codner E, Laven JSE, Teede H (2020). Diabetes: a metabolic and reproductive disorder in women. Lancet Diabetes Endocrinol.

[72] Wakefield LM, Hill CS (2013). Beyond TGFβ: roles of other TGFβ superfamily members in cancer. Nat Rev Cancer.

[73] Wang J, Taylor A, Showeil R, Trivedi P, Horimoto Y, Bagwan I, Ewington L, Lam EWF, El-Bahrawy MA (2014). Expression profiling and significance of VEGF-A, VEGFR2, VEGFR3 and related proteins in endometrial carcinoma. Cytokine.

[74] Whitaker L, Critchley HO (2016). Abnormal uterine bleeding. Best Pract Res Clin Obstet Gynaecol.

[75] Yao Z, Zhang Y, Yan J, Yan L (2021). Deciphering biomarkers of endometriosis by proteomic analysis of eutopic endometrium in infertile patients. J Gynecol Obstet Human Reprod.

[76] Young VJ, Ahmad SF, Duncan WC, Horne AW (2017). The role of TGF-β in the pathophysiology of peritoneal endometriosis. Hum Reprod Updat.

[77] Zhang S, Liu B, Gao L, Mao W, Fu C, Zhang N, Zhang Y, Shen Y, Cao J (2017). Prostaglandin F2α–PTGFR signalling activation, growth factor expression and cell proliferation in bovine endometrial explants. Reprod Fertil Dev.

